# SARS-CoV-2 Infection Among Children in Rondônia, Western Brazilian Amazon: SARS-CoV-2 Among Children in Rondônia

**DOI:** 10.1155/av/6655790

**Published:** 2025-02-17

**Authors:** Gil Guibson Mota Amaral, Gabriella Sgorlon, Valcimar Batista Ferreira, Flávia Serrano Batista, Luana da Silva Soares Farias, Luana Soares Barbagelata, Mirleide Cordeiro dos Santos, Rayssa Layana da Silva Bedran, Deusilene Souza Vieira Dall'Acqua, Najla Benevides Matos

**Affiliations:** ^1^Department of Microorganisms Biology, Oswaldo Cruz Foundation, Fiocruz Rondônia, Porto Velho, Rondônia, Brazil; ^2^Department of Postgraduate Program in Experimental Biology, Federal University of Rondônia-UNIR, Porto Velho, Rondônia, Brazil; ^3^Department of Molecular Virology, Oswaldo Cruz Foundation, Fiocruz Rondônia, Porto Velho, Rondônia, Brazil; ^4^Department of Health Surveillance Agency-AGEVISA, State Coordination of Covid-19, Porto Velho, Rondônia, Brazil; ^5^Department of Virology Section, Evandro Chagas Institute, Ananindeua, Pará, Brazil; ^6^Division of Microbiology, Tropical Medicine Research Center-CEPEM, Porto Velho, Rondônia, Brazil; ^7^National Institute of Epidemiology in Western Amazonia-INCT-EPIAMO, Porto Velho, Rondônia, Brazil

**Keywords:** Brazilian Amazonia, children, COVID-19, epidemiology, SARS-CoV-2

## Abstract

We analyzed 364 children symptomatic or asymptomatic for respiratory symptoms, aged 0.1 month–17 years, selected from primary healthcare units of different municipalities of Rondônia from June 2021 to September 2022. Data were collected from medical and electronic records for epidemiological characterization. The positive cohort (*n* = 96) was quantified using a real-time (RT) qPCR and sequenced by next-generation sequencing. Whole-genome sequences were obtained, SARS-CoV-2 strains were classified using the Pango system, and the maximum likelihood method was used for phylogenetic analyses. Among the patients, 59.34% (216/364) were male and 40.66% (148/364) were female. Children aged 10–14 years showed the highest rate of SARS-CoV-2 positivity. At the time of collection, 54.12% (197/364) of the patients were not age-eligible for immunization against COVID-19. The unvaccinated group accounted for 34.07% (124/364), with the highest proportion in the age groups of 5–9 and 10–14 years. Most patients exhibited mild symptoms. Seventy-nine high-quality genomes were obtained: Delta variant of concern (VOC) was the most prevalent (most abundant strain: AY.99.2), Omicron VOC was reported in 26 individuals (most frequent subvariant: BA.1.1), and Gamma VOC with 22 cases (12 cases of P.1 strain). The viral load showed a median of 7.26 log10 copies/mL, with a mean symptom duration of 4 days. Most of the cases were from children who were unvaccinated and age-ineligible for immunization and were associated with Delta and Omicron VOCs with an increase in subvariants during the study period.

## 1. Introduction

At the onset of the 2019 coronavirus disease (COVID-19) pandemic, patients under the age of 18 years were less susceptible to severe acute respiratory syndrome coronavirus 2 (SARS-CoV-2) infection [1], with lower hospitalization and mortality rates than the adult population [2, 3]. However, this scenario has been changing because of the immunization of most of the adult population and the emergence of new variants of concern (VOCs). Children and adolescents are more exposed to the pathogen, are more vulnerable to SARS-CoV-2 infection, and may be vectors of virus transmission in the community [4–6].

Generally, individuals affected by COVID-19 are asymptomatic. When symptomatic, they exhibit a mild course of the disease without the need for hospitalization [2, 7, 8]. Some patients manifest common symptoms of respiratory infections such as a runny nose, nasal obstruction, cough, and fever [9]. Other severe symptoms have also been reported, including pneumonia, dyspnea, bronchiolitis, and bronchitis and gastrointestinal, neurological, and skin symptoms [10]. In addition, rare and severe disease conditions such as pediatric multisystemic inflammatory syndrome (SIM-P) and long-term sequelae such as long COVID-19 have been reported in this population [11, 12].

COVID-19 global data on the number of cases and deaths among children and adolescents are limited. The COVID-19 dashboard information based on the Coverage database showed that among the 4.4 million COVID-19 deaths, more than 17,400 deaths occurred in individuals under 20 years of age. Of these deaths, 53% occurred among adolescents aged 10–19 years and 47% among children aged 0–9 years [13].

Brazil is one of the countries most affected by the pandemic. According to the Ministry of Health, Brazil has reported 3480 deaths in the population aged 0–19 years since the beginning of the pandemic [14]. The northern region of this country has the highest concentration of children and adolescents [15], and epidemiological studies of childhood respiratory infections by SARS-CoV-2 are rare. This is the first study in Rondônia that evaluates the epidemiology of COVID-19 cases, which is considered indispensable and of priority for pediatric health.

## 2. Subjects, Materials, and Methods

### 2.1. Ethical Aspects and Study Site

This study was conducted at Fiocruz/Rondônia, under the authorization of the FIOCRUZ COVID-19 Genomics Surveillance Network of the Brazilian Ministry of Health and approved by the Research Ethics Committee of the Centro de Pesquisa em Medicina Tropical de Rondônia—CEPEM/RO 4.000.086.

### 2.2. Biological Samples and Epidemiological Data

Samples were collected from 364 individuals, aged 0.1 month–17 years, from reference units for COVID-19 in different municipalities of Rondônia state during June 2021–September 2022. Laboratory diagnosis was performed through RT-qPCR at the Laboratório Central de Saúde Pública de Rondônia (LACEN/RO) and Instituto Evandro Chagas (IEC) and confirmed using the one-step COVID-19 Kit (IBMP, Brazil). Epidemiological data and vaccination profiles were collected from the database records of the Gerenciador de Ambiente Laboratorial (GAL/RO), Sistema de Informação da Vigilância Epidemiológica da Gripe (SIVEP-Gripe), and E-SUS Notifica, and questionnaires with epidemiological data.

### 2.3. Complete Genome Sequencing of SARS-CoV-2

Samples with Ct values of < 25, based on qualitative assays, were selected to allow for high genomic coverage. Nucleotide sequencing was performed using Illumina MiSeq or NextSeq platforms and the COVIDSEQ Kit (Illumina, San Diego, CA, USA) [16].

### 2.4. Data Acquisition and Maximum Likelihood (ML) Phylogeny

High-quality (< 1% N) complete genomes (> 29 kb) of SARS-CoV-2 (*n* = 42) were retrieved from the GISAID EpiCoV database (10.2807/1560–7917. ES.2017.22.13.30494) on October 26, 2021. Sequences from this study (*n* = 1412) and those downloaded from GISAID were aligned using MAFFT (Version 7.487) [17]. The ML method was adopted using IQ-TREE (Version 2.1.3; 10.1093/molbev/msaa015), and the best-fitting nucleotide substitution model was GTR + G + I using the ModelFinder tool [18]. An ultrafast bootstrap with 1000 replicates was used to obtain the branch support values. The tree was visualized and edited using FigTree (Version 1.4.4) [19]. SARS-CoV-2 genomes were classified into lineages using Pangolin software [20], and mutations were analyzed using Nextclade beta [21].

### 2.5. Nucleic Acid Isolation and RT-qPCR

Sample quantification was performed in the Laboratório de Virologia Molecular (FIOCRUZ/RO) and IEC, where viral RNA was extracted from 140 μL of pooled swab samples using a QIAamp Viral RNA Mini Kit (QIAGEN, Germany), according to the manufacturer's instructions. Viral load was determined from 5 μL of extracted viral RNA using the multiplex one-step RT-qPCR assay for SARS-CoV-2 detection, as developed by Queiroz et al. [22].

### 2.6. Statistical Analysis

Descriptive analyses were performed using the central tendencies and dispersion measurements. The chi-square test was used for statistical inference with a significance level of 5% (*p* < 0.05). Statistical analysis was performed, and graphics were generated using *R* (Version 4.0.3) [23].

## 3. Results

All the samples were subjected to RT-qPCR test in order to detect SARS-CoV-2 infection. Patients underwent the test regardless of whether they exhibited symptoms' characteristic of an acute respiratory infection. This measure was adopted to screen the standards of the healthcare units in which the collections were performed, especially during the high peaks of new COVID-19 cases in the state.

**Figure 1:**
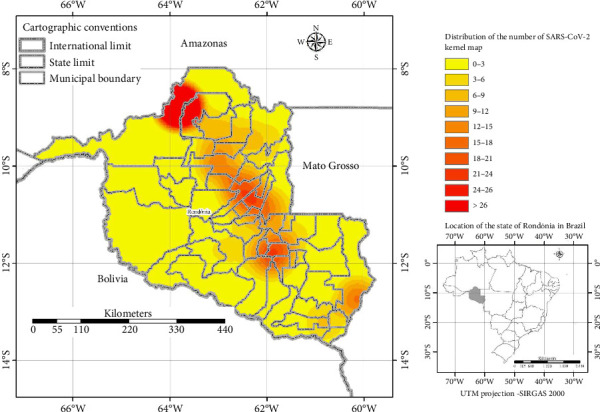
Kernel maps of the Rondônia state, Brazil, showing a spatial distribution and clustering of reported COVID-19 cases among children. The distribution map of positives for SARS-CoV-2 in cities in the Rondônia region. Of these, 26.37% (96/364) tested positive for SARS-CoV-2. The state capital, Porto Velho, had the most expressive numbers with 33.3% (32/96) of cases, followed by Vilhena and Jarú with 7.3% (7/96); Ariquemes with 6.25% (6/96); Ji-Paraná with 6.0% (5/96); Rolim de Moura, Alta Floresta do Oeste, and Candeias do Jamari with 4.2% (4/96) for each municipality; Cerejeiras and Itapuã do Oeste with 3.1% (3/96); Cacoal, Cujubim, Machadinho do Oeste, Ouro Preto do Oeste, and Santa Luzia do Oeste with 2.1% (2/96); and Nova Brasilândia do Oeste, Nova União, São Miguel, Seringueiras, Teixeirópolis, Urupá, Vale do Anari Cacaulândia, Castanheiras, and Guajará Mirim with 1.0% (1/96) of reported cases.

Of these, 26.37% (96/364) tested positive for SARS-CoV-2. The state capital, Porto Velho, had the most expressive numbers with 33.3% (32/96) of cases, followed by Vilhena and Jarú with 7.3% (7/96); Ariquemes with 6.25% (6/96); Ji-Paraná with 6.0% (5/96); Rolim de Moura, Alta Floresta do Oeste, and Candeias do Jamari with 4.2% (4/96) for each municipality; Cerejeiras and Itapuã do Oeste with 3.1% (3/96); Cacoal, Cujubim, Machadinho do Oeste, Ouro Preto do Oeste, and Santa Luzia do Oeste with 2.1% (2/96); and Nova Brasilândia do Oeste, Nova União, São Miguel, Seringueiras, Teixeirópolis, Urupá, Vale do Anari Cacaulândia, Castanheiras, and Guajará Mirim with 1.0% (1/96) of reported cases (Figure 1).

Of the 364 participants, 59.34% (216/364) were male and 40.66% (148/364) were female. A prevalence of positive cases was observed in males, with 65.63% (63/96) recorded, whereas 34.38% (33/96) were in females. In addition, most children aged between 1 and 4 years old (32.7% [119/364]), followed by children 5–9 years old (23.1% [84/364]), < 1 year old (21.4% [78/364]), 10–14 years old (15.4% [56/364]), and > 15 years old (7.4% [27/364]).

Regarding the rate of positive SARS-CoV-2 cases, we observed that individuals between 10 and 14 years of age had the highest positivity rate, corresponding to 30.2% (29/96) of the cases described, followed by patients between 15 and 17 years of age 28.1% (27/96), 5–9 years of age 16.7% (16/96), 1–4 years of age 14.6% (14/96), and children < 1 year of age 10.4% (10/96). Notably, a statistical analysis between age groups and SARS-CoV-2 testing was not possible because no control groups were created for age groups older than 12 years, which may have biased the results for these age groups.

The most frequent symptoms were fever 58.3% (56/96), cough 49.0% (47/96), coryza 41.7% (40/96), headache 37.5% (36/96), sore throat 21.9% (21/96), extra patient-reported symptoms 20.9% (20/96), smell disturbances 4.2% (4/96), and dyspnea and taste disturbances 3.1% (3/96). Furthermore, 4.1% (4/96) of the SARS-CoV-2-positive cases were asymptomatic. The presence or absence of symptoms was analyzed according to the following variables: sex, age group, presence of comorbidities, vaccination, and SARS-CoV-2 positivity. No statistically significant differences were observed in any of the variables (Table 1).

**Figure 2:**
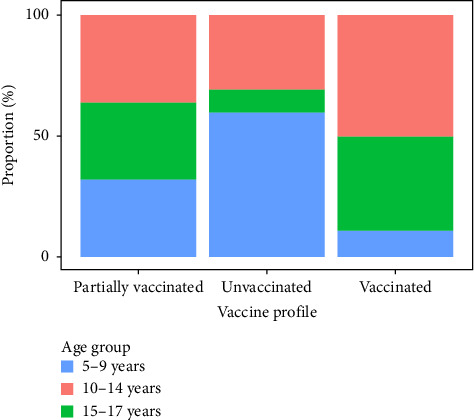
Immunization profile among children in the State of Rondônia. The immunization profile among the children who participated in the study. The immunization profile of the children was not age-eligible for COVID-19 vaccination at the time of collection. The unvaccinated group accounted for 34.07% (124/364), with the highest proportion of children aged 5–9 and 10–14 years. In the group of partially vaccinated individuals, 6.87% (25/364) children had taken only one dose of the immunizer, with the highest number in the 5–9 year-old age group with 32% (8/25), followed by 10–14 years old (36%; 9/26) and 15–17 years old (32%; 8/26). For the vaccinated group (first and second doses), 4.95% (18/364) of the children received complete immunization, and the highest frequency was in the 13–17 year-old age group.

The immunization profile showed that 54.12% (197/364) of the children were not age-eligible for COVID-19 vaccination at the time of collection. The unvaccinated group accounted for 34.07% (124/364), with the highest proportion of children aged 5–9 and 10–14 years. In the group of partially vaccinated individuals, 6.87% (25/364) children had taken only one dose of the immunizer, with the highest number in the 5–9 year-old age group with 32% (8/25), followed by 10–14 years old (36%; 9/26) and 15–17 years old (32%; 8/26). For the vaccinated group (first and second doses), 4.95% (18/364) of the children received complete immunization, and the highest frequency was in the 13–17 year-old age group (Figure 2).

Most positive cases were in individuals not immunized with any dose of the COVID-19 vaccine, which corresponded to 43.75% (42/96) of the cases, followed by individuals in age groups ineligible for vaccination (< 1–4 years old) at 25% (24/96), individuals with an incomplete vaccination comprised 16.67% (16/96), and individuals with a complete vaccination comprised 14.58% (14/96) of positive cases. Among the 96 samples analyzed, 79 were sequenced by next-generation sequencing, which presented good quality for genomic profile analysis, where the prevalence of three variants of SARS-CoV-2 in the population aged 0–17 years could be observed. In this cohort, the Delta variant had the highest number of reported cases (39.24%; 31/79), followed by Omicron (32.91%; 26/79) and Gamma (27.85%; 22/79).

**Figure 3:**
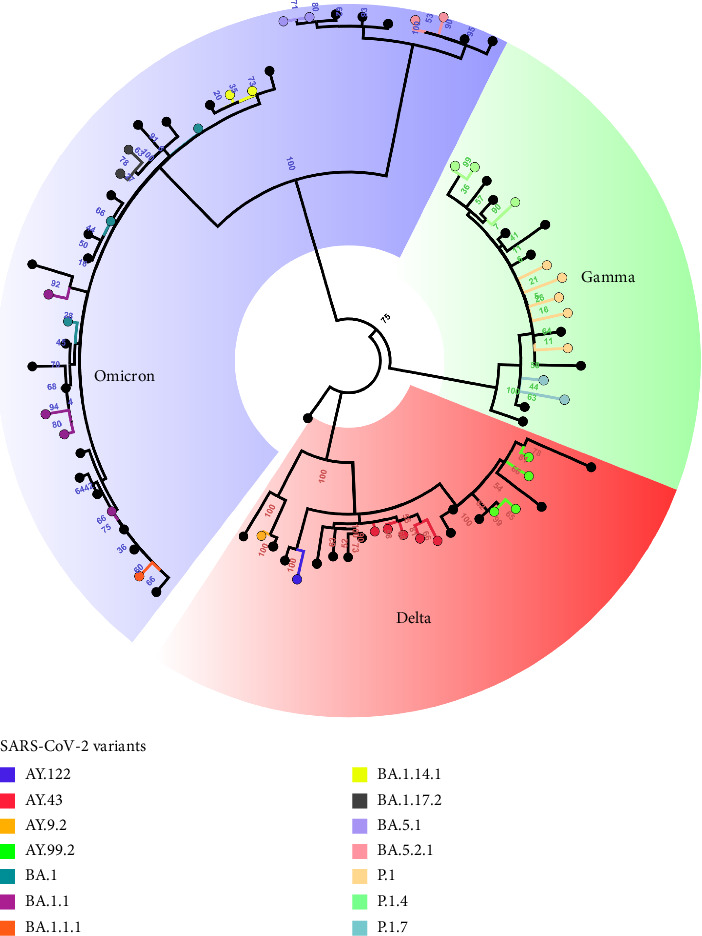
Representation of the distribution of Delta, Gamma, and Omicron variants of SARS-CoV-2 among children. The distribution of variants circulating in the Rondônia region. The Gamma variant had only three subvariants, where P.1 had the highest proportion, then P.1.4, and P.1.7 of the cases. The Delta variant had four circulating subvariants in this cohort: AY.99.2 of cases, followed by AY.43, AY.122, and AY.9.2. The Omicron variant had the most subvariants, and seven representatives were identified. BA.1.1.

The Gamma variant had only three subvariants, where P.1 had the highest proportion (15.19%; 12/79), then P.1.4 (7.59%; 6/79), and P.1.7 (5.06%; 4/79) of the cases. The Delta variant had four circulating subvariants in this cohort: AY.99.2 with 18.99% (15/79) of cases, followed by AY.43 (16.46%; 13/79), AY.122 (2.53%; 2/79), and AY.9.2 (1.27%; 1/79). The Omicron variant had the most subvariants, and seven representatives were identified. BA.1.1 had 10.13% (8/79) of the cases; BA.1 and BA.1.1.1 had 6.33% (5/79) each; BA.1.14.1 with 3.80% (3/79); BA.1.17.2 and BA.5.1 with 2.53% (2/79) each; and BA.5.2.1 with 1.27% (1/79) of the cases (Figure 3).

**Figure 4:**
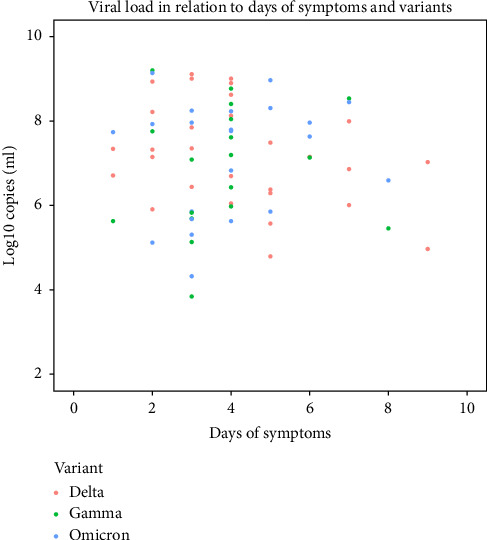
Viral load measured by the number of days after the onset of symptoms. The viral load status related to the period of symptoms and distribution between variants. The viral load in individuals < 18 years of age characterized with the variants showed a median of 7.26 log10 copies/mL with an average 4 days of symptoms (min: 1 day and max: 9 days). Two children aged 14 and 6 years showed quantifiable viral load after 9 days of symptoms, both with VOC Delta infection, and another two under the age of 2 years showed quantifiable viral load after 8 days of symptoms, one with VOC Omicron and other with VOC Gamma infection.

The viral load in individuals < 18 years of age characterized with the variants showed a median of 7.26 log10 copies/mL with an average 4 days of symptoms (min: 1 day and max: 9 days). Two children aged 14 and 6 years showed quantifiable viral load after 9 days of symptoms, both with VOC Delta infection, and another two under the age of 2 years showed quantifiable viral load after 8 days of symptoms, one with VOC Omicron and other with VOC Gamma infection (Figure 4).

## 4. Discussion

Brazil is one of the countries most affected by the COVID-19 pandemic with a high number of cases and disproportionate deaths in five regions of the country. Previous studies have revealed that the northern states of the country accounted for the maximum number of deaths from COVID-19 in the pediatric population [24]. In this scenario, we conducted the first study in the state of Rondônia involving children and adolescents aged 0–17 years to investigate the clinical and epidemiological aspects of the disease in these individuals.

According to our results, 26.37% of the patients admitted in the study tested positive for SARS-CoV-2 infection, with progression from asymptomatic to moderate cases of infection. These data corroborate those of an Indian study that evaluated the behavior of this population during the two waves of COVID-19 infection in the country. The results showed a low percentage of positive cases during both periods, with 14.7% (711/4821) in the first wave and a slight increase in the second wave (21.2%; 759/3583) [25]. Although we observed a low percentage of positive cases, the number of infected children has increased significantly since the beginning of the COVID-19 pandemic. This may be because the testing criteria for SARS-CoV-2 have changed as the risk of exposure, symptoms associated with COVID-19, laboratory testing capacity, and priority populations evolved throughout the pandemic [10].

Remarkably, most individuals in this cohort were symptomatic and presented with mild symptoms. These data contrast with those of studies conducted at the beginning and during the pandemic, which reported that the vast majority of the study population was asymptomatic for COVID-19 [2, 26, 27].

The clinical condition observed in symptomatic cases concurred with large studies conducted in the pediatric population [28, 29], in which patients under 18 years of age usually present with mild and nonspecific symptoms; fever was the only symptom that affected half of the cases, followed by cough [8, 30, 31] and coryza [32] as the most frequent symptoms. No statistical relationship was observed between the age of the patients and symptoms presented, although previous studies have reported that children under 5 years of age were more likely to present with severe clinical conditions of the disease [2].

Notably, a large part of the population studied presented with symptoms' characteristic of respiratory infections and tested negative for SARS-CoV-2 infection. Several pathogens can cause respiratory infections, most of which have a viral etiology, such [33, 34] as influenza virus, respiratory syncytial virus, and rhinovirus. Several etiological studies have described the pediatric population as one of the main transmitters of these viruses in the community, with the elderly being the most affected [34]. These results indicate the importance of using tools to detect the most frequent pathogens associated with respiratory infections and the use of molecular tools, such as real-time PCR, that allow a more economical, rapid, and sensitive test [34–36]. Using such tools rectifies indiscriminate use of antibiotics, administration of specific antivirals, and the use of strategies to control the spread of the pathogen [37].

In this study, the Gamma, Delta, and Omicron VOCs were found to be associated with infections in children (< 12 years) and young people (12–17 years), with Delta and Omicron showing more expressive numbers. After the emergence of the Delta and Omicron variants of SARS-CoV-2, high rates of infection and hospitalization among children have been reported [38, 39]. However, a severity profile was not observed among the infected patients.

In addition to new variants, the accumulation of mutations has led to an increase in subvariant strains over time, raising concerns regarding vaccine escape and phenotypic changes in SARS-CoV-2 infections in at-risk populations [40–42]. The results presented here show an increase in subvariants since Gamma infections, relative to Delta and Omicron infections, which were subsequently characterized in this study.

No differences were noted in the medians of the quantified viral loads among the VOC characterized in this study. The mean viral load detection in relation to symptom duration was the standard for SARS-CoV-2 [43]. These data demonstrate that children reach high viral loads and are therefore important conductors of the spread of SARS-CoV-2 in the general population, as was observed in a study on the respiratory syncytial virus in which children with high viral loads were more likely to transmit the virus [44].

In conclusion, this study verified the behavior of SARS-CoV-2 in children and adolescents in the state of Rondônia. Most positive cases were associated with Delta and Omicron VOCs, and an increase in the subvariants was observed during the study period. The clinical manifestations were mild and showed good evolution. Although the population studied is affected to a lesser degree by the new coronavirus when compared to older populations, children and adolescents can reach high levels of viral load, even if not associated with the severity of the disease. As observed in this study, these individuals can contribute to the dissemination of the virus in the community. Furthermore, the highest rate of positive cases was in patients who had not been immunized against COVID-19, followed by those who were age-ineligible for immunization. The importance of immunization in these age groups is reinforced because most of the adult population has completed the vaccination schedule, and these individuals may become targets of the pathogen, enabling an increase in the number of cases.

## Figures and Tables

**Table 1:** Sociodemographic and clinical features of pediatrics patients.

Features	Symptomatic	%	Asymptomatic	%	ORc (CI 95%)
Gender					
Female	144	97.3	4	2.7	1.563 (0.426–7.082)
Male	207	95.8	9	4.2	
Age					
< 1	75	96.2	3	3.8	1
1–4	115	96.6	4	3.4	0.869 (0.189–3.95)
5–9	80	95.2	4	5.4	1.249 (0.189–3.995)
10–14	55	98.2	1	1.8	0.454 (0.460–4.487)
15–17	29	96.3	12	3.7	0.707 (0.016–5.044)
Comorbidity					
No	314	96.3	12	3.7	0.707 (0.016–5.044)
Yes	37	97.4	1	2.6	
Vaccine					
Ineligible age	189	96.4	7	3.6	1
Unvaccinated	119	96.0	5	4.0	1.134 (3.656)
Vaccinated	43	97.7	1	2.3	0.627 (0.075–5.237)
SARS-CoV-2 infection					
Negative	259	96.6	9	3.4	0.274 (0.274–4.614)
Positive	92	95.8	4	4.2	

## Data Availability

The entire dataset supporting the results of this study is available on request from the corresponding author.
